# Validation of nutritional screening tools in patients undergoing cancer surgery in low- and middle-income countries

**DOI:** 10.3389/fnut.2025.1576916

**Published:** 2025-06-05

**Authors:** Jana Sremanakova, Stephen R. Knight, Marie Carmela M. Lapitan, Edwin Yenli, Stephen Tabiri, Dhruva Ghosh, Pamela A. Kingsley, Apple Valparaiso, Ewen M. Harrison, Maria Thomas, Parvez D. Haque, Sreejith K. Veetil, Atul Suroy, Ashish Choudhrie, Rohin Mittal, Rajkumar Kottayasamy Seenivasagam, Bipradas Roy, Debra Jones, Sorrel T. Burden

**Affiliations:** ^1^Faculty of Medicine, Biology and Health, Manchester Academic Health Science Centre, University of Manchester, Manchester, United Kingdom; ^2^Centre for Medical Informatics, Usher Institute, University of Edinburgh, Edinburgh, United Kingdom; ^3^Health Economics and Health Technology Assessment, School of Health and Wellbeing, University of Glasgow, Glasgow, United Kingdom; ^4^Institute of Clinical Epidemiology, National Institutes of Health, University of the Philippines Manila, Manila, Philippines; ^5^Department of Surgery, School of Medicine, University for Development Studies, Tamale, Ghana; ^6^Department of Paediatric Surgery, Christian Medical College, Ludhiana, India; ^7^Department of Radiation Oncology, Christian Medical College, Ludhiana, India; ^8^Department of Surgery, Philippine General Hospital, University of the Philippines, Manila, Philippines; ^9^Department of Biochemistry, Christian Medical College, Ludhiana, India; ^10^Department of General Surgery, Christian Medical College and Hospital, Ludhiana, India; ^11^Padhar Hospital, Betul, India; ^12^Christian Medical College, Vellore, India; ^13^PSG Institute of Medical Sciences and Research, Coimbatore, India; ^14^Tata Medical Centre, Kolkata, India; ^15^Intestinal Failure Unit, Salford Royal NHS Foundation Trust, Manchester, United Kingdom

**Keywords:** malnutrition, low and middle income countries, nutritional screening tools, surgical patients, Malnutrition Universal Screening Tool, MUST, Patient Generated Subjective Global Assessment

## Abstract

Approximately one-third of patients are severely malnourished prior to surgery in low- and middle-income countries (LMICs). Identifying the most appropriate tool for detecting malnutrition is a critical first step toward enabling effective treatment interventions. Therefore, this study aimed to assess the validity and reliability of nutritional screening tools in patients with cancer scheduled for surgery in LMICs. Participants included adults undergoing either curative elective or palliative surgeries in Ghana, India, and the Philippines. Nutritional status was assessed using anthropometric measurements, the Malnutrition Universal Screening Tool (MUST), and the Patient-Generated Subjective Global Assessment (PG-SGA). Data were analysed using Bland–Altman plots with confidence intervals (CIs) and intra-class correlation coefficients (ICCs) to assess inter-rater reliability. Sensitivity and specificity tests were conducted using the Area Under the Receiver Operating Characteristics Curve (AUROC). A total of 167 participants were recruited, with a mean age of 53.3 years (SD 14.7) and a mean body mass index (BMI) of 23.0 kg/m^2^ (SD 4.9). The proportion of participants identified as at risk of malnutrition was 53.3% using MUST, 47.3% using PG-SGA SF, and 66% using the full PG-SGA. When compared to the PG-SGA, MUST and PG-SGA SF had AUROCs of 0.78 (95% CI: 0.73–0.87) and 0.76 (95% CI: 0.68–0.83), respectively. MUST demonstrated a sensitivity of 85% and a specificity of 25%, while PG-SGA SF showed a sensitivity of 93% and a specificity of 42%. Excellent inter-rater agreement was observed for anthropometric measurements, with ICC values >0.9 across all assessments. Both MUST and PG-SGA SF demonstrated good sensitivity when compared to PG-SGA. However, PG-SGA SF demonstrated slightly greater specificity than MUST. Based on these findings, PG-SGA SF is recommended for preoperative nutritional screening in LMICs.

## 1 Introduction

Undernutrition has been recognised as a global health issue and a key priority within the United Nations 2030 Agenda for Sustainable Development (1). The burden of undernutrition is disproportionately higher in low- and middle-income countries (LMICs) compared to high-income countries, with preoperative prevalence rates ranging from 50 to 80% (2, 3). This translates to as many as three in five patients being malnourished before surgery (4). Cancer surgery patients are particularly vulnerable, often experiencing complications such as dysphagia, anorexia, sarcopenia, cachexia, and malnutrition (5). These challenges highlight the urgency of addressing malnutrition during the perioperative period in LMICs to enable timely and appropriate nutritional interventions. Despite the substantial burden, the evidence base is heavily biased toward research conducted in high-income settings, which accounts for approximately 90% of existing studies (6). Addressing perioperative malnutrition in LMICs has been identified as a high-priority research area by surgical experts working in lower-resource settings (7).

A large prospective cohort study conducted by the Global Surgical Collaboration (8) found that one-third of patients undergoing surgery for cancer were severely malnourished when assessed using Global Leadership Initiative on Malnutrition (GLIM) criteria (9). Additionally, severe malnutrition was independently associated with 30-day mortality and surgical site infections (10). When malnutrition has been attenuated preoperatively with dietary advice and oral nutritional supplements, improved early outcomes following cancer surgery have been demonstrated (11, 12). Furthermore, a systematic review found that preoperative nutritional support consistently reduced surgical morbidity and mortality across different surgical populations in LMICs (13).

Therefore, identifying patients at risk of malnutrition is essential before surgery to enable the commencement of nutritional support preoperatively. There is a lack of data on the validity and reliability of nutritional screening tools in LMICs. A high level of knowledge regarding screening for malnutrition was observed among healthcare professionals in LMICs, as indicated by semi-structured interviews. However, a lack of financial resources, robust operational systems, and policies was identified as a barrier to optimal nutritional care (14). The Subjective Global Assessment (SGA) was the most frequently used tool to assess malnutrition (15), followed by the Malnutrition Universal Screening Tool (MUST) (16), the Nutritional Risk Index (NRI) (17), and the Patient-Generated Subjective Global Assessment (PG-SGA) (18).

In the absence of a gold standard for assessing malnutrition, assessment and screening tools are highly dependent on the patient population and care setting. Although SGA is considered the most appropriate tool for use in patients with cancer in hospital settings (19), PG-SGA has been suggested as a suitable tool for patients with cancer undergoing surgery (20–22). The PG-SGA is a subjective, relatively lengthy, and time-intensive tool that requires clinical expertise, making it less convenient for routine use. For effective screening, a quick tool that can be applied with minimal training across diverse clinical settings is essential. Hence, the short form, PG-SGA SF, which can be used as a standalone tool to assess the risk of malnutrition, represents an easier and more convenient tool. The short form of PG-SGA can be completed by patients or healthcare professionals, and it has been previously validated in oncological settings as a sensitive and reliable tool to detect malnutrition (23–25). The validation study showed that the sensitivity and specificity criteria are met at a score of ≥2 (27). Alternatively, the MUST is another quick and straightforward tool that requires minimal clinical judgement as it includes weight and height, and it was designed to be performed by different professionals in all settings, including hospitals and the community (26). All PG-SGA, PG-SGA SF, and MUST meet the GLIM criteria recently proposed as a core diagnostic criterion for malnutrition in adults in clinical settings (9).

Several studies validated the PG-SGA SF against the PG-SGA in high-income countries (23, 24, 27), but in LMICs, evidence is currently lacking. Furthermore, only a few studies have validated PG-SGA against the MUST in high-income countries (24, 25, 28, 29), while there is a paucity of studies performed in LMICs. However, one study conducted in China validated MUST and the PG-SGA against the GLIM criteria (30). Identifying the most appropriate and validated tool for detecting malnutrition in low- and middle-income countries (LMICs) is the first step required to conduct effective interventions addressing malnutrition. This study aimed to validate nutritional screening tools to identify malnutrition in patients with cancer scheduled for surgery in LMICs.

## 2 Materials and methods

### 2.1 Study design and population

The study aimed to assess the validity and reliability of nutritional screening tools for identifying malnutrition in LMICs. The Standards for Reporting Diagnostic Accuracy studies (STARD) were used to ensure the completeness and transparency of reporting (31).

Participants were adults undergoing curative or palliative elective cancer surgery. Participants were recruited from eight hospitals in Ghana (*n* = 2), India (*n* = 5), and the Philippines (*n* = 1) between June 2020 and April 2022. Patients under 16 years of age, those undergoing emergency surgery, those with suspected benign pathology, or those unable to provide informed consent were excluded.

### 2.2 Ethical approval

All study centres received local regulatory approval prior to the commencement of the study. Ethical approval was gained from the University of Edinburgh (UoE) Ethics Committee on 7th October 2019.

### 2.3 Data collection

Assessments were completed by independent healthcare professionals at two time points, 3 h apart, and professionals were blinded to the previous assessment completed. As the study was integrated into routine clinical practice, it was not feasible for all hospital participants to be assessed by the same professional or for the same professionals to conduct repeated assessments at both time points.

To prevent measurement error, anthropometric assessments were conducted with standardised and calibrated instruments at each site. The equipment was provided locally, where possible or supplied by the UoE. The anthropometric measurements included were as follows:

- Height and weight
- Recall unintentional weight loss in the preceding 3–6 months
- Waist circumference (cm)
- Mid-upper arm circumference (MUAC) (cm)
- Mid-upper muscle circumference (MAMC) (cm),
- Triceps skin-fold thickness (TSF) (mm)
- Handgrip strength (kg force units).

Waist circumference, MUAC, and TSF were repeatedly measured for accuracy. Standard operating procedures were provided to each hospital to ensure consistency and internal validity in anthropometric assessment (Supplementary material 1). Furthermore, the proportion of food eaten at mealtimes was recorded as none, a quarter, half, three-quarters, or all of the meals. Serum albumin and C-reactive protein were recorded if taken as part of routine care. The PG-SGA tool and the MUST tool were completed. Local investigators uploaded records to a secure website using the Research Electronic Data Capture system (32).

### 2.4 Malnutrition screening tool calculation

The PG-SGA (27) is a tool that evaluates nutritional information through two components: Part A, known as the PG-SGA Short Form (PG-SGA SF), is completed by the patient, while Part B is completed by a healthcare professional. Based on the assessment, patients are classified into one of three categories: (A) well nourished, (B) suspected malnutrition, or (C) severely malnourished. In addition to categorical classification, the PG-SGA provides a total numerical score that guides the levels of required intervention. A score of 0–1 indicates that no intervention is required; a score of 2–3 suggests that patient and family education may be beneficial; a score of 4–8 warrants intervention by a dietitian; and a score above 9 indicates a critical need for improved symptom management and/or intensive nutritional support.

Part A of the PG-SGA SF can be used independently to assess the risk of malnutrition. It is scored on a numerical scale with a maximum score of 35 (23). Based on the score, patients are categorised as “well-nourished” (score 0–1), “at risk of malnutrition” (score 2–8), or “severely malnourished” (score >9) (23).

For the MUST, a score of 0 indicates a low risk of malnutrition, a score of 1 indicates a medium risk, and a score ≥2 or more indicates a high risk of malnutrition (16).

### 2.5 Data analysis

Data were summarised using descriptive statistics and measures of variance. Inter-rater reliability was assessed to compare the consistency of nutritional assessment and screening tools reported as continuous variables, using Bland–Altman plots with confidence intervals (CIs) and intra-class correlation coefficients (ICC). To evaluate the accuracy of the PG-SGA, PG-SGA SF, and MUST tools, sensitivity and specificity analyses were performed and evaluated using the Area Under the Receiver Operating Characteristic Curve (AUROC). An AUROC value of 0.5 indicates no predictive ability, 0.8 is considered good, and 1.0 represents perfect discrimination (33). Scores from the MUST, PG-SGA, and PG-SGA SF tools were dichotomised into two categories: “no risk” and “at risk” of malnutrition to calculate sensitivity and specificity. Participants classified as “well nourished” (PG-SGA A), with a MUST score of 0, or a PG-SGA SF score of 0–1, were categorised as “not at risk.” Those classified as PG-SGA B or C, with MUST scores of≥1, or PG-SGA SF scores of≥2, were classified as “at risk.” Statistical analyses were performed using IBM SPSS Statistics (Version 23, Chicago, USA), and results were considered statistically significant at a *p*-value of <0.05.

## 3 Results

### 3.1 Population characteristics

In total, 167 patients were recruited: 15 from Ghana (9%), 101 from India (60.5%), and 51 from the Philippines (30.5%). The mean age was 53.3 years (SD 14.7) and ranged from 18 to 87 (Table 1). More women than men were recruited for the study. The most common cancer site was the gastrointestinal tract (*n* = 85, 50.9%), followed by head and neck (*n* = 24, 14.4%) and breast cancers (*n* = 17, 10.2%). The majority of patients had American Society of Anaesthesiology (ASA) scores of 1 and 2 (*n* = 149, 89.2%), and Eastern Cooperative Oncology Group (ECOG) performance status of 0 and 1 (*n* = 148, 88.6%).

**Table 1:** Population characteristics

Characteristics	Total	Ghana	India	Philippines
Sample size, n (%)	167	15 (9)	101 (60.5)	51 (30.5)
Age, Mean (SD)	53.3 (14.7)	57.2 (22.8)	52.4 (13.9)	53.9 (13.3)
Gender, Male/Female ratio	78/89	5/10	49/52	24/27
Disease location, n (%)				
Breast	17 (10.2)	5 (33.4)	8 (7.9)	4 (7.8)
Fibrosarcoma	5 (3.0)	0 (0.0)	0 (0.0)	5 (9.8)
Gastrointestinal tract	85 (50.9)	6 (40.0)	49 (48.5)	30 (58.8)
Gynaecological	15 (9.0)	2 (13.3)	12 (11.9)	1 (2.0)
Head and neck	24 (14.4)	0 (0.0)	21 (20.8)	3 (5.9)
Lungs	3 (1.8)	0 (0.0)	3 (3.0)	0 (0.0)
Thyroid	1 (0.6)	0 (0.0)	1 (1.0)	0 (0.0)
Upper urinary tract	11 (6.6)	0 (0.0)	4 (4.0)	6 (11.8)
Urological	6 (3.7)	2 (13.3)	3 (3.0)	2 (3.9)
ECOG, n (%)				
0 - Fully active	96 (57.41)	8 (53.3)	55 (54.5)	33 (64.7)
1 - Restricted in physical strenuous activity only	52 (31.14)	4 (26.67)	35 (34.7)	13 (25.5)
2 - Ambulatory and capable of all self-care	16 (9.58)	1 (6.67)	10 (9.9)	5 (9.8)
3 - Capable of only limited self-care	3 (1.80)	2 (13.3)	1 (1.0)	0 (0.0)
ASA, n (%)				
I (normal/healthy)	47 (28.14)	2 (13.33)	36 (35.6)	9 (17.6)
II (mild systemic disease)	102 (61.08)	4 (26.67)	60 (59.4)	38 (74.5)
III (severe systemic disease)	10 (5.99)	1 (6.67)	5 (5.0)	4 (7.8)
IV (severe systemic disease, threat to life)	1 (0.60)	1 (6.67)	0 (0.0)	0 (0.0)
Missing	7 (4.19)	7 (46.67)	0 (0.0)	0 (0.0)
Albumin*, Mean (SD)	37.5 (8.0)	30.25 (8.0)	38.7 (7.9)	36.1 (7.8)
CRP*, Mean (SD)	18.3 (23.8)	Na	17.4 (23.9)	27.0 (29.7)
Diabetes, n (%)				
No	148 (88.8)	14 (93.3)	85 (87.1)	45 (88.2)
Yes	19 (11.4)	1 (6.7)	13 (12.9)	6 (11.8)
Smoking status, n (%)				
Never smoked	129 (77.3)	15 (100.0)	79 (78.2)	36 (70.6)
Past smoker	27 (16.2)	0 (0.0)	14 (13.9)	12 (24.0)
Current smoker	9 (5.3)	0 (0.0)	6 (5.9)	3 (5.4)
Missing	2 (1.2)	0 (0.0)	2 (2.0)	0 (0.0)

* Preoperatively; ASA - American Society of Anaesthesiology score, CRP – C reactive protein, ECOG – Eastern Cooperative Oncology Group performance status, SD – standard deviation.

### 3.2 Nutritional assessment

The majority of assessments were completed by doctors (70%), followed by nurses (20%), and dietitians or nutritionists (10%). The majority of participants reported consuming all their meals (70%), 10% consumed three-quarters, 10% had half, and 10% consumed one-quarter or less of their meals. The mean weight of participants was 59.4 kg (SD 12.8) and ranged between 30 kg and 104 kg. The mean BMI was 23.0 kg/m^2^ (SD 4.9), with values ranging from 12.3 to 41 kg/m^2^. Differences between measurements at the first and second assessment were small (*p* > 0.1 for all outcomes) (see Table 2).

**Table 2:** Anthropometric measurements conducted independently, 3 h apart.

Measurement	*N*	First assessment		Second assessment		Mean difference			t-test
		Mean (SD)	Range	Mean (SD)	Range	MD	SE	95%CI	*p* value
BMI	167	23.0 (4.9)	12.3–41.0	23.0 (4.9)	12.3–41.0	0	0.5	−1.1 to 1.1	0.7
Handgrip left	167	20.9 (9.2)	1.5–53.0	21.0 (9.3)	1.2–54.0	0.1	1.0	−1.9 to 2.1	0.8
Handgrip right	167	21.4 (9.4)	1.6–60.0	21.6 (9.3)	2.0–60.0	0.2	1.0	−1.8 to 2.2	0.3
Height	167	1.60 (0.1)	1.4–1.8	1.6 (0.1)	1.4–1.8	0	0	−0.02 to 0.02	0.8
MAMC	167	22.1 (4.3)	4.3–34.8	22.3 (4.1)	6.1–37.8	0.2	0.5	−0.7 to 1.1	0.9
MUAC*	167	26.8 (4.5)	9.5–41.4	26.8 (4.5)	9.75–39.1	0.0	0.5	−1.0 to 1.0	0.3
Tricpes skinfold*	167	14.8 (8.9)	4.0–60.7	14.4 (7.7)	4.0–42.3	−0.4	0.9	−2.2 to 1.4	0.2
Waist circum*	167	87.7 (14.0)	35.75–130.0	88.0 (13.8)	36.0–130.5	0.3	1.5	−2.7 to 3.3	0.1
Weight	167	59.4 (12.8)	30.0–104.6	59.4 (12.8)	30.0–104.5	0	1.4	−2.8 to 2.8	1

*Average of repeated measures, *N*, Total numbers; Circum, circumference; MAMC, Mid-upper muscle circumference; MUAC, Mid-upper arm circumference; MD, Mean difference; SE, Standard error; SD, Standard deviation, circumference.

**Figure 1:**
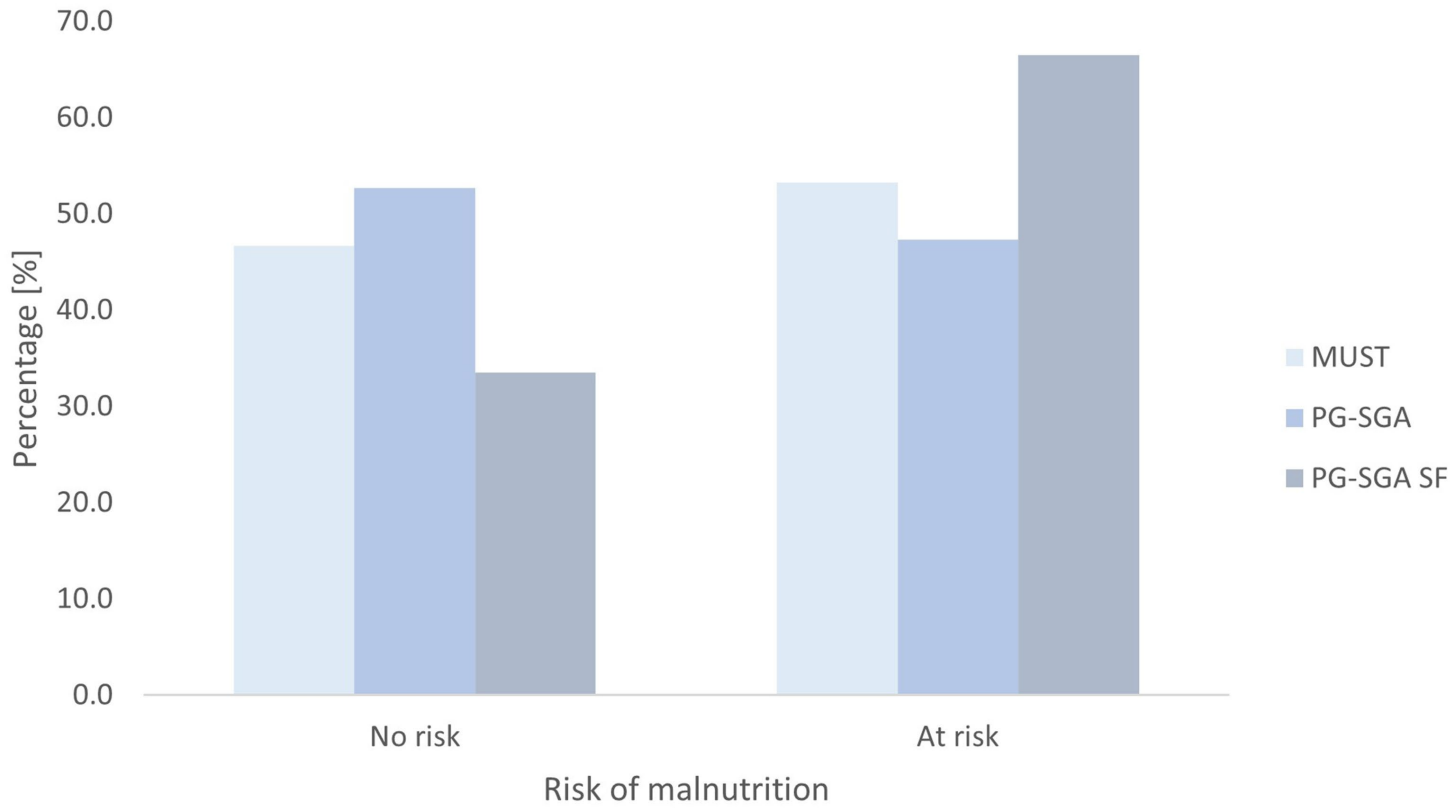
Nutritional status assessed by dichotomised Malnutrition Universal Screening Tool (MUST), Patient-Generated Subjective Global Assessment (PG-SGA), and Patient-Generated Subjective Global Assessment Short Form (PG-SGA SF) tool.

According to the PG-SGA tool, 88 participants (52.7%) were assessed as low risk (A), 49 participants (29.3%) were medium risk (B), and 30 participants (18%) were high risk (C). Based on PG-SGA SF, 56 participants (33.5%) were assessed as A, 54 participants (32.3%) were B, and 57 participants (34.1%) were C. According to MUST, 78 participants (46.7%) were assessed as being low risk, 34 participants (20.4%) were identified as medium risk, and 55 (32.9%) were identified as being at high risk of malnutrition. Details are presented in Table 3. When the scores were dichotomised, minor differences were observed between PG SGA and MUST. In contrast, the PG-SGA SF tool identified 18.7% more participants at risk than the PG SGA tool and 12.7% more than the MUST tool (Figure 1).

**Table 3:** Nutritional status assessed by Malnutrition Universal Screening Tool (MUST), Patient-Generated Subjective Global Assessment (PG-SGA), and Patient-Generated Subjective Global Assessment Short Form (PG-SGA SF) tool independently, 3 h apart.

Assessment tool	MUST				PG-SGA					PG-SGA SF				
Assessment time	First assessment		Second assessment			First assessment		Second assessment			First assessment		Second assessment	
Score	*n*	[%]	*n*	[%]	Score	*n*	[%]	*n*	[%]	Score	*n*	[%]	*n*	[%]
Low risk	78	46.7	79	47.3	Stage A	88	52.7	86	51.5	Stage A	56	33.5	55	32.9
Medium risk	34	20.4	33	19.8	Stage B	49	29.3	53	31.7	Stage B	54	32.3	56	33.5
High risk	55	32.9	54	32.3	Stage C	30	18.0	28	16.8	Stage C	57	34.1	56	33.5

*n*, subtotal numbers; MUST, Malnutrition Universal Screening Tool; PG-SGA, Patient-Generated Subjective Global Assessment; PG-SGA SF, Patient-Generated Subjective Global Assessment short form; %, percentage.

### 3.3 Reliability of assessment

The intra-class correlation coefficient was above 0.9 for all assessment methods (BMI, 1.0, 95% CI 1.0 to 1.0; MUAC, 0.99, 95% CI 0.99 to 0.99; MAMC, 0.96, 95% CI 0.95 to 0.97; TSF, 0.92, 95% CI 0.90 to 0.94; left handgrip, 0.96, 95% CI 0.97 to 0.98; right handgrip strength, 0.98, 95% CI 0.97 to 0.98; PG-SGA SF, 0.97, 95% CI 0.96 to 0.98). These results indicate excellent inter-rater agreement among clinical staff. Bland–Altman plots and regression analysis showed good agreement and no proportional bias for MUAC (ß 0.012, *p* = 0.48), BMI (ß 0.005, *p* = 0.52), handgrip strength right (ß 0.009, *p* = 0.69), and left (ß − 0.008, *p* = 0.77); however, proportional bias was shown for MAMC (ß 0.067, *p* = 0.03), TSF (ß 0.158, *p* = 0.0001), and PG-SGA SF (ß 0.06, *p* = 0.03) (Supplementary material 2).

### 3.4 Specificity and sensitivity testing

**Figure 2:**
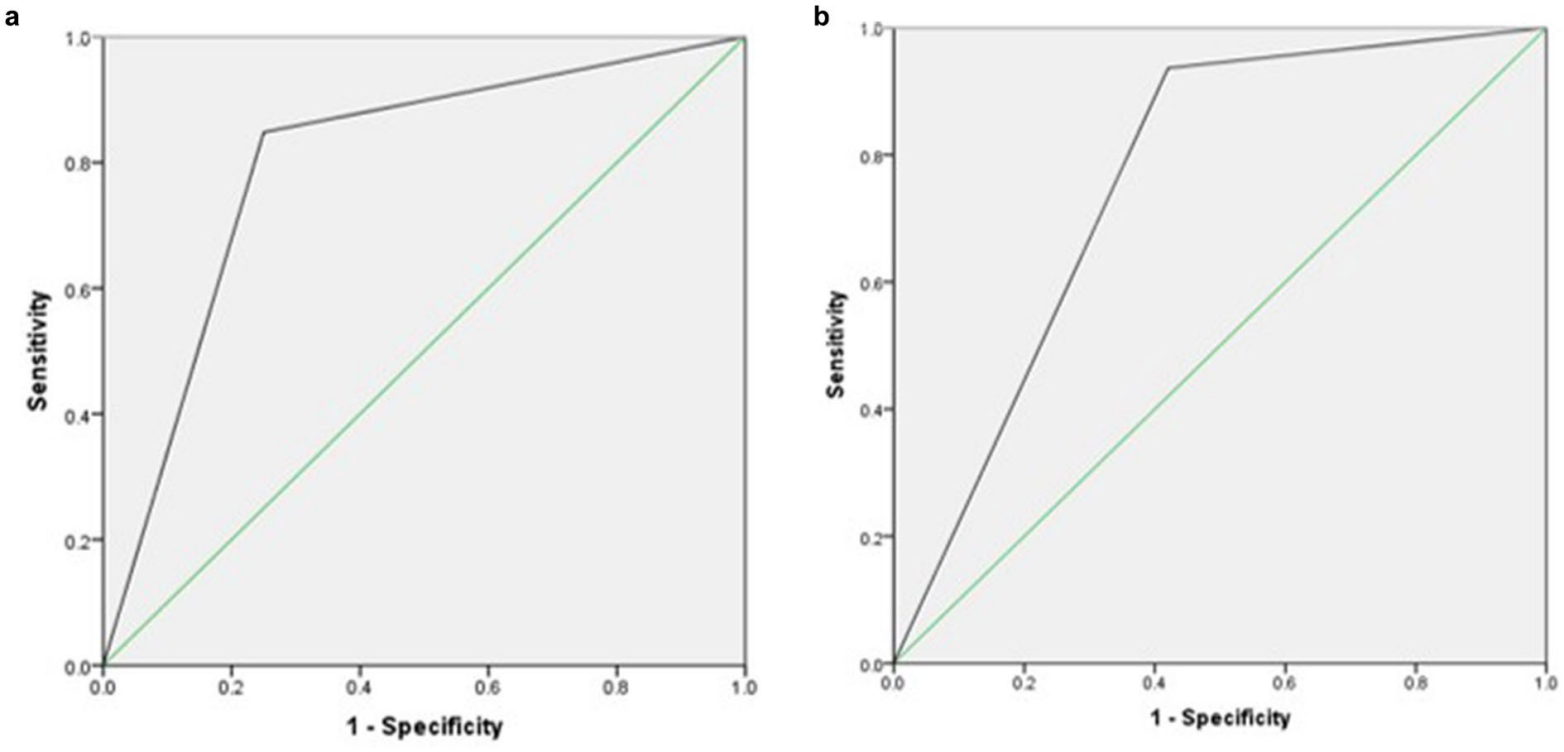
**(a)** Receiver operating curve agreement between Malnutrition Universal Screening Tool (MUST) and Patient-Generated Subjective Global Assessment (PG-SGA). **(b)** Receiver operating curve agreement between Patient-Generated Subjective Global Assessment Short Form (PG-SGA SF) and Patient-Generated Subjective Global Assessment (PG-SGA).

Table 4 and Figure 2 describe the relationship between MUST, PG-SGA, and PG-SGA SF. Compared to PG-SGA, MUST exhibited a sensitivity of 85%, a specificity of 25%, and an AUC of 0.79, indicating good agreement. When PG-SGA SF was compared with PG-SGA, the sensitivity was 93%, the specificity was 42%, and the AUC was 0.76, indicating good agreement.

**Table 4:** Results between Malnutrition Universal Screening Tool (MUST), Patient-Generated Subjective Global Assessment (PG-SGA), and Patient-Generated Subjective Global Assessment Short Form (PG-SGA SF), respectively.

Assessment tool	AUC	95%CI	Sensitivity	Specificity
MUST	0.79	0.73–0.87	0.85	0.25
PG-SGA SF	0.76	0.68–0.83	0.93	0.42

AUC, Area under the curve; CI, Confidence Interval; MUST, Malnutrition Universal Screening Tool; PG-SGA SF, Patient-Generated Subjective Global Assessment Short Form.

## 4 Discussion

This study aimed to validate nutrition screening tools used to assess malnutrition in patients undergoing elective surgery for cancer in LMICs. The study demonstrated the validity of MUST and PG-SGA SF tools for detecting malnutrition compared to a criterion measure, PG-SGA. Both tools demonstrated high sensitivity but low specificity, indicating that while they effectively identify malnutrition when present, they may be less accurate in identifying individuals without malnutrition. However, the tools are not as robust at detecting people who are not malnourished. The use of MUST, in particular, across clinical settings may be susceptible to false-positive results, given its low specificity of 25%. This could impact clinical decision-making, whereby patients who are not at risk of malnutrition may be incorrectly classified as requiring nutritional intervention, which could lead to inappropriate treatment, inefficient resource allocation, and misaligned clinical priorities. However, the difference in AUC on the Bland–Altman plots between the MUST and PG-SGA SF is small (0.79 vs. 0.76), suggesting comparable performance of both methods in identifying malnutrition; hence, the difference in specificity (25% vs. 42%) may not be considered important clinically, suggesting that the observed variation may not meaningfully impact the practical application of these methods in clinical settings. Anthropometric measurements showed excellent inter-rater agreement among healthcare professionals, indicating strong reproducibility, particularly for MUAC, BMI, and handgrip strength.

Previously, only a few studies have validated nutritional screening tools in patients undergoing surgery for cancer (23, 27, 34), with studies within LMICs lacking. These findings support the use of MUST and PG-SGA SF as screening tools to detect the risk of malnutrition prior to elective cancer surgery. Similar results were reported on the validity of MUST against PG-SGA in hospitalised patients, where MUST was shown to have good specificity and sensitivity to detect malnutrition (25). Moreover, previously, the MUST demonstrated a fair to good agreement with the Mini Nutritional Assessment and an excellent agreement with the Nutrition Risk Score and Subjective Global Assessment (35). In patients undergoing radiotherapy, the MUST has been shown to be a simple screening method with a high validity for early screening and identification of patients where further nutritional interventions are required (34).

These findings differ from those reported by Neto et al. (28) in a validation study involving hospitalised patients. Neto et al. (28) observed that PG-SGA was more sensitive to malnutrition risk than MUST (28). In this study, MUST identified fewer patients at medium risk of malnutrition (20.4% vs. 32.9%) compared to PG-SGA, but more patients at high risk of malnutrition (29.3% vs. 18.0%). A possible explanation for the differences may be related to the measurements and observations used in the tools. MUST uses BMI and unplanned weight loss rather than other factors that contribute to malnutrition, including percentage of oral intake or functionality, which can affect nutritional intake. In contrast, the PG-SGA and the PG-SGA SF take into consideration these additional factors, including eating pattern, symptoms, and disease state.

A specific subgroup of overweight and obese individuals suffering from malnutrition warrants attention, particularly those with sarcopenic obesity, a condition that is often highly prevalent in cancer patients (36). Identifying these individuals as malnourished can be problematic with screening tools that only incorporate unintentional weight loss and weight-for-height measurements (29). In addition, there is an ongoing debate regarding cut-off points used for BMI in Asian populations and the international classification for BMI in different ethnic groups (37). Hence, using BMI to assess malnutrition in Asian ethnic groups may introduce bias if body proportions and areas of fat deposition are not considered (38, 39).

The PG-SGA SF was the most sensitive tool in our study, which identified 32.3% of patients at medium risk of malnutrition and 34% at high risk. This is consistent with findings that PG-SGA SF shows better sensitivity and diagnostic accuracy than MUST (27). These findings are aligned with results from another study showing that 37% of participants classified by MUST as being at low risk of malnutrition were identified as medium or high risk by PG-SGA SF (24). In contrast to the MUST, the PG-SGA SF consistently demonstrates excellent validity and sensitivity in detecting the risk of malnutrition compared to the reference method of PG-SGA (23, 27) in cancer patients undergoing chemotherapy. Although both MUST and PG-SGA SF are predictive of mortality when compared to the reference standard PG-SGA, PG-SGA SF was shown to better predict malnutrition and worse outcomes in hospitalised patients (24).

The main strength of the current study was that the nutritional assessment and screening tools were implemented in routine clinical practice across multiple centres in three low- and middle-income countries (LMICs). Also, the nutritional assessment and screening tools were completed twice, and healthcare professionals were blinded to the previous assessment. To our knowledge, this is the first study that validated nutritional screening tools in LMICs in the cancer population. Limitations include fewer patients undergoing cancer surgery in Ghana and restrictions due to the COVID-19 pandemic that influenced our ability to recruit patients to the study. Furthermore, a possible explanation for PG-SGA identifying fewer participants at risk of malnutrition compared to other tools could be related to the subjective judgements required for PG-SGA, which could differ across countries.

In conclusion, MUST and PG-SGA SF show good agreement with the full PG-SGA assessment and are thus recommended for screening malnutrition risk in LMICs. This study highlights the practical advantages of PG-SGA SF over MUST in these settings. However, further studies should be conducted in a larger population of patients scheduled for cancer surgery to support our findings.

## Data Availability

The raw data supporting the conclusions of this article will be made available by the authors without undue reservation.
