# Supplementary material for: Validation of nutritional screening tools in patients undergoing cancer surgery in low- and middle-income countries
Source: Front Nutr. 2025 Jun 5;12:1576916. doi: 10.3389/fnut.2025.1576916 (PMC12176562; doi:10.3389/fnut.2025.1576916)
Supplement: Supplementary file 1 [file Table_1.docx]

Supplementary Material

**Supplementary material 1**

**Standard Operation Procedures**

**CASE REPORT FORM**

| **Patient details** | |
| --- | --- |
| Patient ID |  |
| Age (years) |  |
| Gender | Male / Female / Unknown |
| **Diagnosis & malnutrition screening** | |
| Cancer type |  |
| Essential TNM stage |  |
| Date of malnutrition assessment | dd/mm/yyyy |
| Height (cm) |  |
| Weight (kg) |  |
| Previous weight in last 3-6 months (kg) |  |
| Current meal consumption | All / three quarters / half / quarter / nothing |
| Hand grip strength (kg) |  |
| Waist circumference (cm) |  |
| Mid-upper arm circumference (cm) |  |
| Triceps skin-fold thickness (cm) |  |
| Serum albumin (g/L) |  |
| CRP (mg/L) |  |
| Malnutrition screening tool score (SGA / MUST)  PERSON 1 | Individual components of tool placed here |
| Malnutrition screening tool score (SGA / MUST)  PERSON 2 | Individual components of tool placed here |
| **Operation** | |
| Date and time of admission | dd/mm/yyyy |
| Date and time of operation | dd/mm/yyyy |
| Surgical intent | Curative / Palliative |
| Operative approach | Laparoscopic  Laparoscopic converted to open  Open |
| Primary operation performed |  |
| **Outcomes** | |
| Length of post-operative stay (days) |  |
| How was 30-day follow-up status achieved? | Still in-patient OR readmitted  Clinic review  Telephone review  Community / home review  Discharged before 30-day follow-up and not contacted again |
| 30-day mortality | Alive / Dead / Unknown |
| Date of death | dd/mm/yyyy |
| Post-operative complication (Clavien-Dindo 1 or 2) | Yes / No / Unknown |
| Post-operative complication (Clavien-Dindo 3 or 4) | Yes / No / Unknown |
| 30-day unplanned hospital readmission | Yes / No / Unknown |
| Surgical site infection | Yes / No / Unknown |

**Height**

Equipment: stadiometer

Height to the nearest Cm

- Shoes removed
- Participants stands straight and looks ahead with back facing the height measure tool (stadiometer)
- Lower the height measure to rest on top of participants head
- Take measurement reading to the nearest centimetre (cm) and record in metres (m) (i.e.: 170cm = 1.70m)

**Body weight**

Equipment: scales

- Shoes removed
- Check for and remove any other heavy items such as belt, items in pockets etc.
- Participant stands upright on the scales and looks straight ahead
- Participant should not touch or lean on anything else whilst measure is taken
- Ensure participant is standing still then take measurement in kilograms(kg) to the nearest 0.1kg

**Preparation for Mid upper arm circumference & Triceps skinfold**

The upper arm muscle circumference and the triceps skinfold can be used to measure the quantity of fat and muscle mass in the body. This is a surrogate measure useful when a person cannot be weighed or if their weight is not likely to be a true reflection of the persons’ actual weight, e.g. if the patient has oedema or ascites.

This is the preparation step to be carried out before measuring mid upper arm circumference and triceps skinfold

Equipment: flexible tape measure

Determine and mark the mid-point between the shoulder and elbow (using left arm):

- Find the pointy part of your shoulder bone and mark it with a small dot or cross.
- Find the point part on the left top edge of your radius and mark it (alternatively, bend the elbow so that your hand is close to your shoulder and then mark the top left point of your elbow)
- Take a tape and measure distance between your two marked points. Then mark the mid-point (e.g. distance is 35 cm, mark the skin at 17.5 cm)
- The area where you marked you mid-point will inform your horizontal line at which you will be measuring triceps skinfold and mid arm circumference

**Mid upper arm circumference**

The upper arm muscle circumference and the triceps skinfold can be used to measure the quantity of fat and muscle mass in the body. This is a surrogate measure useful when a person cannot be weighed or if their weight is not likely to be a true reflection of the persons’ actual weight, e.g. if the patient has oedema or ascites.

Equipment: flexible tape measure

Mid upper arm circ. (MUAC)

- Ask the patient to stand straight with the arm hanging by the side and the hand in the mid-prone position
- Place the measuring tape gently around the arm where you have marked the point
- Make sure that tape is horizontal (same on both sides of the arm). Do not pull or tighten the tape
- Record the number on tape measure and repeat the measurement second time
- Record both on REDCap

**Triceps skinfolds**

The upper arm muscle circumference and the triceps skinfold can be used to measure the quantity of fat and muscle mass in the body. This is a surrogate measure useful when a person cannot be weighed or if their weight is not likely to be a true reflection of the persons’ actual weight, e.g. if the patient has oedema or ascites.

Equipment: flexible tape measure and skinfold calipers

Triceps skinfolds (TS)

- Ask the patient to stand straight with the arm hanging by the side and the hand in the mid-prone position
- Place the measuring tape gently around the arm where you have marked the mid-point of the arm. Make sure that tape is horizontal (same on both sides of the arm)
- Use tape to make a small mark line at the back of the arm where the triceps are
- Then make a vertical mark to cross the horizontal mark to be in the middle of the back of the arm. You have created the cross mark where you measure triceps skinfold
- Place your finger and the thumb on marked cross on the back of the arm
- Hold the skinfold in a vertical position with one hand Use other hand to grab the skinfold with the calipers
- Let go of the handgrip and wait two seconds before reading the value
- Repeat this measurement three times
- Calculate the mean of the three measurements

**Calculating Mid arm muscle circumference**

MAMC is a surrogate measure of fat free mass and is calculated using MUAC and TSF.

Equipment: calculator

Mid arm muscle circ. (MAMC)

The formula for calculating MAMC is:

MAMC (cm) = MUAC (cm) – 3.14 x TSF (cm)

Centile tables allow assessment of changes in total body muscle mass over time.

This will be done on REDCAP

**Hand grip strength**

Hand grip strength is a measure of skeletal muscle strength.

Equipment: Dynamometer

- The Participant should self-adjust the dynamometer so it fits comfortably in their hand
- Prior to data collection allow the participant to have a trial session with the dynamometer
- The participant should stand up with both arms pending sideways to the body with the dynamometer facing outwards away from the body
- Instruct the participant to grip the dynamometer with maximum strength
- Perform 1 trial on each hand
- Record the value for each side

**Supplementary material 2 Bland and Altman plots**

|  | | | | | | | | |  | |  | |  | | | |  | | |  | |  | |  | |  | |  | |  |
| --- | --- | --- | --- | --- | --- | --- | --- | --- | --- | --- | --- | --- | --- | --- | --- | --- | --- | --- | --- | --- | --- | --- | --- | --- | --- | --- | --- | --- | --- | --- |
|  | |  | |  | |  | |  | |  | |  | |  | | | |  | | |  | |  | |  | |  | |  | |
| 1. Body Mass Index (BMI) | |  | |  | |  | |  | |  | |  | |  | | | |  | | |  | |  | |  | |  | |  | |
| 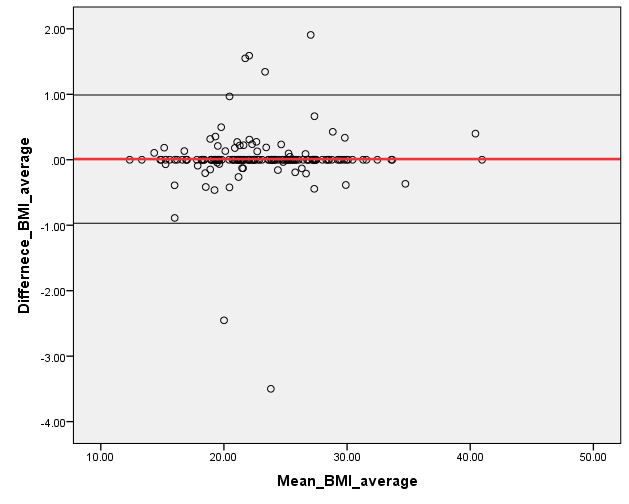   \|  \| \| --- \| | |  | |  | |  | |  | |  | |  | |  | | | |  | | |  | |  | |  | |  | |  | |
|  | |  | |  | |  | |  | |  | |  | |  | | | |  | | |  | |  | |  | |  | |  | |
|  | |  | |  | |  | |  | |  | |  | |  | | | |  | | |  | |  | |  | |  | |  | |
|  | |  | |  | |  | |  | |  | |  | |  | | | |  | | |  | |  | |  | |  | |  | |
|  | |  | |  | |  | |  | |  | |  | |  | | | |  | | |  | |  | |  | |  | |  | |
|  | |  | |  | |  | |  | |  | |  | |  | | | |  | | |  | |  | |  | |  | |  | |
|  | |  | |  | |  | |  | |  | |  | |  | | | |  | | |  | |  | |  | |  | |  | |
|  | |  | |  | |  | |  | |  | |  | |  | | | |  | | |  | |  | |  | |  | |  | |
|  | |  | |  | |  | |  | |  | |  | |  | | | |  | | |  | |  | |  | |  | |  | |
|  | |  | |  | |  | |  | |  | |  | |  | | | |  | | |  | |  | |  | |  | |  | |
|  | |  | |  | |  | |  | |  | |  | |  | | | |  | | |  | |  | |  | |  | |  | |
|  | |  | |  | |  | |  | |  | |  | |  | | | |  | | |  | |  | |  | |  | |  | |
|  | |  | |  | |  | |  | |  | |  | |  | | | |  | | |  | |  | |  | |  | |  | |
|  | |  | |  | |  | |  | |  | |  | |  | | | |  | | |  | |  | |  | |  | |  | |
|  | |  | |  | |  | |  | |  | |  | |  | | | |  | | |  | |  | |  | |  | |  | |
|  | |  | |  | |  | |  | |  | |  | |  | | | |  | | |  | |  | |  | |  | |  | |
|  | |  | |  | |  | |  | |  | |  | |  | | | |  | | |  | |  | |  | |  | |  | |
|  | |  | |  | |  | |  | |  | |  | |  | | | |  | | |  | |  | |  | |  | |  | |
|  | |  | |  | |  | |  | |  | |  | |  | | | |  | | |  | |  | |  | |  | |  | |
|  | |  | |  | |  | |  | |  | |  | |  | | | |  | | |  | |  | |  | |  | |  | |
|  | |  | |  | |  | |  | |  | |  | |  | | | |  | | |  | |  | |  | |  | |  | |
|  | |  | |  | |  | |  | |  | |  | |  | | | |  | | |  | |  | |  | |  | |  | |
|  | |  | |  | |  | |  | |  | |  | |  | | | |  | | |  | |  | |  | |  | |  | |
| 1. Mid-upper arm circumference (MUAC) | |  | |  | |  | |  | |  | |  | |  | | | |  | | |  | |  | |  | |  | |  | |
|  | |  | |  | |  | |  | |  | |  | |  | | | |  | | |  | |  | |  | |  | |  | |
|  | |  | |  | |  | |  | |  | |  | |  | | | |  | | |  | |  | |  | |  | |  | |
|  | |  | |  | |  | |  | |  | |  | |  | | | |  | | |  | |  | |  | |  | |  | |
|  | |  | |  | |  | |  | |  | |  | |  | | | |  | | |  | |  | |  | |  | |  | |
|  | |  | |  | |  | |  | |  | |  | |  | | | |  | | |  | |  | |  | |  | |  | |
| 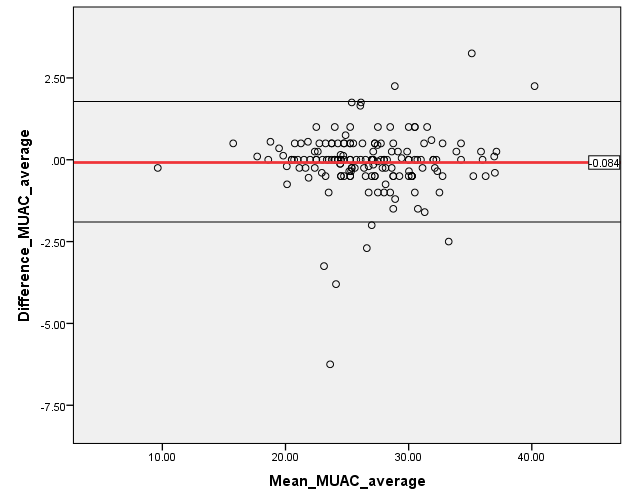   \|  \| \| --- \| | |  | |  | |  | |  | |  | |  | |  | | | |  | | |  | |  | |  | |  | |  | |
|  | |  | |  | |  | |  | |  | |  | |  | | | |  | | |  | |  | |  | |  | |  | |
|  | |  | |  | |  | |  | |  | |  | |  | | | |  | | |  | |  | |  | |  | |  | |
|  | |  | |  | |  | |  | |  | |  | |  | | | |  | | |  | |  | |  | |  | |  | |
|  | |  | |  | |  | |  | |  | |  | |  | | | |  | | |  | |  | |  | |  | |  | |
|  | |  | |  | |  | |  | |  | |  | |  | | | |  | | |  | |  | |  | |  | |  | |
|  | |  | |  | |  | |  | |  | |  | |  | | | |  | | |  | |  | |  | |  | |  | |
|  | |  | |  | |  | |  | |  | |  | |  | | | |  | | |  | |  | |  | |  | |  | |
|  | |  | |  | |  | |  | |  | |  | |  | | | |  | | |  | |  | |  | |  | |  | |
|  | |  | |  | |  | |  | |  | |  | |  | | | |  | | |  | |  | |  | |  | |  | |
|  | |  | |  | |  | |  | |  | |  | |  | | | |  | | |  | |  | |  | |  | |  | |
|  | |  | |  | |  | |  | |  | |  | |  | | | |  | | |  | |  | |  | |  | |  | |
| 1. Mid-upper muscle circumference (MAMC) | |  | |  | |  | |  | |  | |  | |  | | | |  | | |  | |  | |  | |  | |  | |
|  | |  | |  | |  | |  | |  | |  | |  | | | |  | | |  | |  | |  | |  | |  | |
| 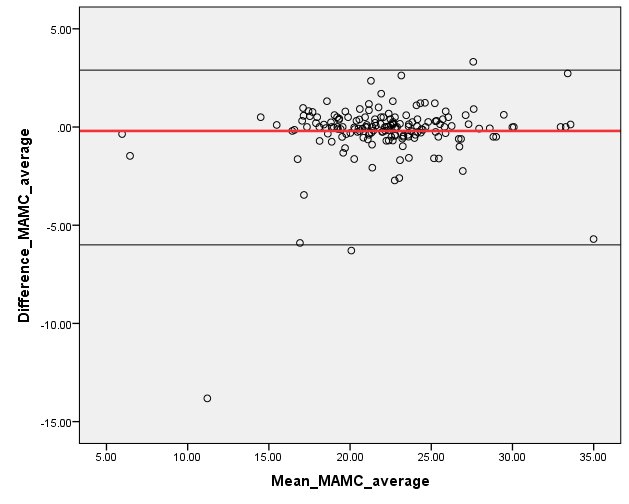 | |  | |  | |  | |  | |  | |  | |  | | | |  | | |  | |  | |  | |  | |  | |
|  | |  | |  | |  | |  | |  | |  | |  | | | |  | | |  | |  | |  | |  | |  | |
|  | |  | |  | |  | |  | |  | |  | |  | | | |  | | |  | |  | |  | |  | |  | |
|  | |  | |  | |  | |  | |  | |  | |  | | | |  | | |  | |  | |  | |  | |  | |
|  | |  | |  | |  | |  | |  | |  | |  | | | |  | | |  | |  | |  | |  | |  | |
|  | |  | |  | |  | |  | |  | |  | |  | | | |  | | |  | |  | |  | |  | |  | |
|  | |  | |  | |  | |  | |  | |  | |  | | | |  | | |  | |  | |  | |  | |  | |
|  | |  | |  | |  | |  | |  | |  | |  | | | |  | | |  | |  | |  | |  | |  | |
|  | |  | |  | |  | |  | |  | |  | |  | | | |  | | |  | |  | |  | |  | |  | |
|  | |  | |  | |  | |  | |  | |  | |  | | | |  | | |  | |  | |  | |  | |  | |
|  | |  | |  | |  | |  | |  | |  | |  | | | |  | | |  | |  | |  | |  | |  | |
|  | |  | |  | |  | |  | |  | |  | |  | | | |  | | |  | |  | |  | |  | |  | |
|  | |  | |  | |  | |  | |  | |  | |  | | | |  | | |  | |  | |  | |  | |  | |
|  | |  | |  | |  | |  | |  | |  | |  | | | |  | | |  | |  | |  | |  | |  | |
| \|  \| \| --- \| | |  | |  | |  | |  | |  | |  | |  | | | |  | | |  | |  | |  | |  | |  | |
|  | |  | |  | |  | |  | |  | |  | |  | | | |  | | |  | |  | |  | |  | |  | |
|  | |  | |  | |  | |  | |  | |  | |  | | | |  | | |  | |  | |  | |  | |  | |
|  | |  | |  | |  | |  | |  | |  | |  | | | |  | | |  | |  | |  | |  | |  | |
|  | |  | |  | |  | |  | |  | |  | |  | | | |  | | |  | |  | |  | |  | |  | |
|  | |  | |  | |  | |  | |  | |  | |  | | | |  | | |  | |  | |  | |  | |  | |
|  | |  | |  | |  | |  | |  | |  | |  | | | |  | | |  | |  | |  | |  | |  | |
| 1. Triceps skin-fold thickness (TRF) | |  | |  | |  | |  | |  | |  | |  | | | |  | | |  | |  | |  | |  | |  | |
|  | |  | |  | |  | |  | |  | |  | |  | | | |  | | |  | |  | |  | |  | |  | |
| 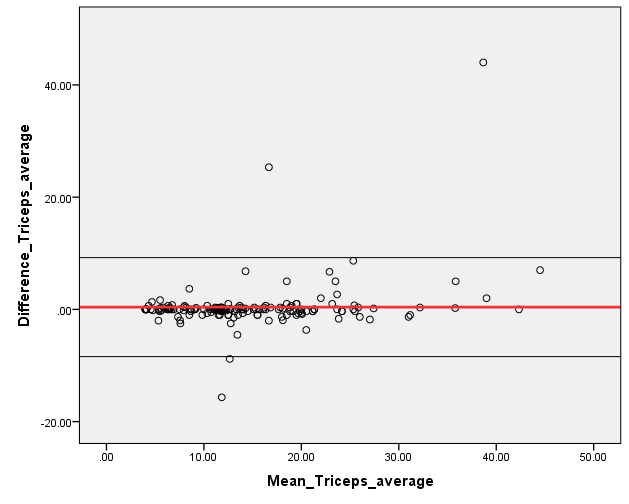 | |  | |  | |  | |  | |  | |  | |  | | | |  | | |  | |  | |  | |  | |  | |
|  | |  | |  | |  | |  | |  | |  | |  | | | |  | | |  | |  | |  | |  | |  | |
|  | |  | |  | |  | |  | |  | |  | |  | | | |  | | |  | |  | |  | |  | |  | |
|  | |  | |  | |  | |  | |  | |  | |  | | | |  | | |  | |  | |  | |  | |  | |
|  | |  | |  | |  | |  | |  | |  | |  | | | |  | | |  | |  | |  | |  | |  | |
|  | |  | |  | |  | |  | |  | |  | |  | | | |  | | |  | |  | |  | |  | |  | |
|  | |  | |  | |  | |  | |  | |  | |  | | | |  | | |  | |  | |  | |  | |  | |
|  | |  | |  | |  | |  | |  | |  | |  | | | |  | | |  | |  | |  | |  | |  | |
|  | |  | |  | |  | |  | |  | |  | |  | | | |  | | |  | |  | |  | |  | |  | |
|  | |  | |  | |  | |  | |  | |  | |  | | | |  | | |  | |  | |  | |  | |  | |
|  | |  | |  | |  | |  | |  | |  | |  | | | |  | | |  | |  | |  | |  | |  | |
|  | |  | |  | |  | |  | |  | |  | |  | | | |  | | |  | |  | |  | |  | |  | |
|  | |  | |  | |  | |  | |  | |  | |  | | | |  | | |  | |  | |  | |  | |  | |
|  | |  | |  | |  | |  | |  | |  | |  | | | |  | | |  | |  | |  | |  | |  | |
|  | |  | |  | |  | |  | |  | |  | |  | | | |  | | |  | |  | |  | |  | |  | |
|  | | |  | |  | |  | |  | |  | |  | | | |  | | |  | |  | |  | |  | |  | |  |
| 1. Handgrip – right | |  | | | | | | | | | | | | | | | | | | | | | | | | |  | |  | |
|  | |  |  |  |  |  |  |  |  |  |  |  |  |  |  |  |  |  |  |  |  |  |  |  |  |  |  | |  | |
|  | |  |  |  |  |  |  |  |  |  |  |  |  |  |  |  |  |  |  |  |  |  |  |  |  |  |  | |  | |
|  | |  |  |  |  |  |  |  |  |  |  |  |  |  |  |  |  |  |  |  |  |  |  |  |  |  |  | |  | |
|  | |  |  |  |  |  |  |  |  |  |  |  |  |  |  |  |  |  |  |  |  |  |  |  |  |  |  | |  | |
|  | |  |  |  |  |  |  |  |  |  |  |  |  |  |  |  |  |  |  |  |  |  |  |  |  |  |  | |  | |
|  | |  |  |  |  |  |  |  |  |  |  |  |  |  |  |  |  |  |  |  |  |  |  |  |  |  |  | |  | |
|  | |  |  |  |  |  |  |  |  |  |  |  |  |  |  |  |  |  |  |  |  |  |  |  |  |  |  | |  | |
|  | |  |  |  |  |  |  |  |  |  |  |  |  |  |  |  |  |  |  |  |  |  |  |  |  |  |  | |  | |
|  | |  |  |  |  |  |  |  |  |  |  |  |  |  |  |  |  |  |  |  |  |  |  |  |  |  |  | |  | |
|  | |  |  |  |  |  |  |  |  |  |  |  |  |  |  |  |  |  |  |  |  |  |  |  |  |  |  | |  | |
| 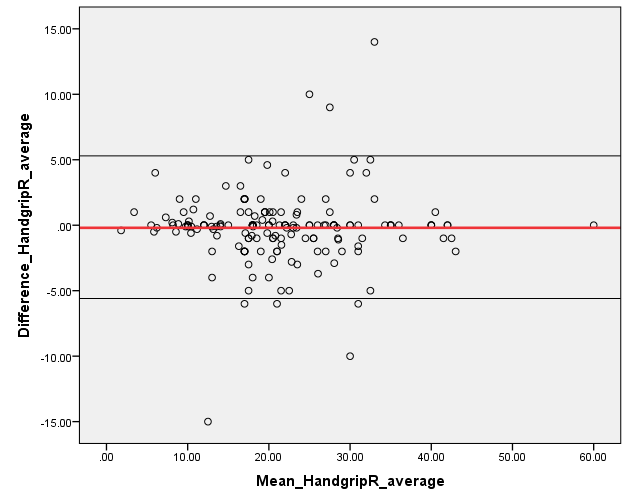 | |  |  |  |  |  |  |  |  |  |  |  |  |  |  |  |  |  |  |  |  |  |  |  |  |  |  | |  | |
|  | |  |  |  |  |  |  |  |  |  |  |  |  |  |  |  |  |  |  |  |  |  |  |  |  |  |  | |  | |
|  | |  |  |  |  |  |  |  |  |  |  |  |  |  |  |  |  |  |  |  |  |  |  |  |  |  |  | |  | |
|  | |  |  |  |  |  |  |  |  |  |  |  |  |  |  |  |  |  |  |  |  |  |  |  |  |  |  | |  | |
|  | |  |  |  |  |  |  |  |  |  |  |  |  |  |  |  |  |  |  |  |  |  |  |  |  |  |  | |  | |
|  | |  |  |  |  |  |  |  |  |  |  |  |  |  |  |  |  |  |  |  |  |  |  |  |  |  |  | |  | |
|  | |  |  |  |  |  |  |  |  |  |  |  |  |  |  |  |  |  |  |  |  |  |  |  |  |  |  | |  | |
|  | |  |  |  |  |  |  |  |  |  |  |  |  |  |  |  |  |  |  |  |  |  |  |  |  |  |  | |  | |
|  | |  |  |  |  |  |  |  |  |  |  |  |  |  |  |  |  |  |  |  |  |  |  |  |  |  |  | |  | |
|  | | | | | | | | | | | | | | |  |  | | |  |  |  |  |  |  |  |  |  |  |  |  |
|  |  |  |  |  |  |  |  |  |  |  |  |  |  |  |  |  | | |  |  |  |  |  |  |  |  |  |  |  |  |
|  |  |  |  |  |  |  |  |  |  |  |  |  |  |  |  |  | | |  |  |  |  |  |  |  |  |  |  |  |  |
|  |  |  |  |  |  |  |  |  |  |  |  |  |  |  |  |  | | |  |  |  |  |  |  |  |  |  |  |  |  |
| 1. Handgrip – left | |  | |  | |  | |  | |  | |  | |  | | | |  | | |  | |  | |  | |  | |  | |
|  | |  | |  | |  | |  | |  | |  | |  | | | |  | | |  | |  | |  | |  | |  | |
|  | | |  | |  | |  | |  | |  | |  | | | |  | | |  | |  | |  | |  | |  | |  |
|  | |  | | | | | | | | | | | | | | | | | | | | | | | | |  | |  | |
|  | |  |  |  |  |  |  |  |  |  |  |  |  |  |  |  |  |  |  |  |  |  |  |  |  |  |  | |  | |
|  | |  |  |  |  |  |  |  |  |  |  |  |  |  |  |  |  |  |  |  |  |  |  |  |  |  |  | |  | |
|  | |  |  |  |  |  |  |  |  |  |  |  |  |  |  |  |  |  |  |  |  |  |  |  |  |  |  | |  | |
| 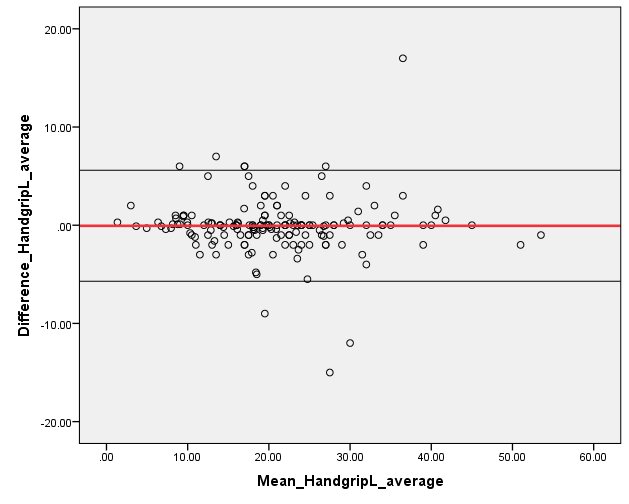 | |  |  |  |  |  |  |  |  |  |  |  |  |  |  |  |  |  |  |  |  |  |  |  |  |  |  | |  | |
|  | |  |  |  |  |  |  |  |  |  |  |  |  |  |  |  |  |  |  |  |  |  |  |  |  |  |  | |  | |
|  | |  |  |  |  |  |  |  |  |  |  |  |  |  |  |  |  |  |  |  |  |  |  |  |  |  |  | |  | |
|  | |  |  |  |  |  |  |  |  |  |  |  |  |  |  |  |  |  |  |  |  |  |  |  |  |  |  | |  | |
|  | |  |  |  |  |  |  |  |  |  |  |  |  |  |  |  |  |  |  |  |  |  |  |  |  |  |  | |  | |
|  | |  |  |  |  |  |  |  |  |  |  |  |  |  |  |  |  |  |  |  |  |  |  |  |  |  |  | |  | |
|  | |  |  |  |  |  |  |  |  |  |  |  |  |  |  |  |  |  |  |  |  |  |  |  |  |  |  | |  | |
|  | |  |  |  |  |  |  |  |  |  |  |  |  |  |  |  |  |  |  |  |  |  |  |  |  |  |  | |  | |
|  | |  |  |  |  |  |  |  |  |  |  |  |  |  |  |  |  |  |  |  |  |  |  |  |  |  |  | |  | |
|  | |  |  |  |  |  |  |  |  |  |  |  |  |  |  |  |  |  |  |  |  |  |  |  |  |  |  | |  | |
|  | |  |  |  |  |  |  |  |  |  |  |  |  |  |  |  |  |  |  |  |  |  |  |  |  |  |  | |  | |
|  | |  |  |  |  |  |  |  |  |  |  |  |  |  |  |  |  |  |  |  |  |  |  |  |  |  |  | |  | |
|  | |  |  |  |  |  |  |  |  |  |  |  |  |  |  |  |  |  |  |  |  |  |  |  |  |  |  | |  | |
| 1. Patient Generated Subjective Global Assessment (PG-SGA) | |  |  |  |  |  |  |  |  |  |  |  |  |  |  |  |  |  |  |  |  |  |  |  |  |  |  | |  | |
|  | |  |  |  |  |  |  |  |  |  |  |  |  |  |  |  |  |  |  |  |  |  |  |  |  |  |  | |  | |
| 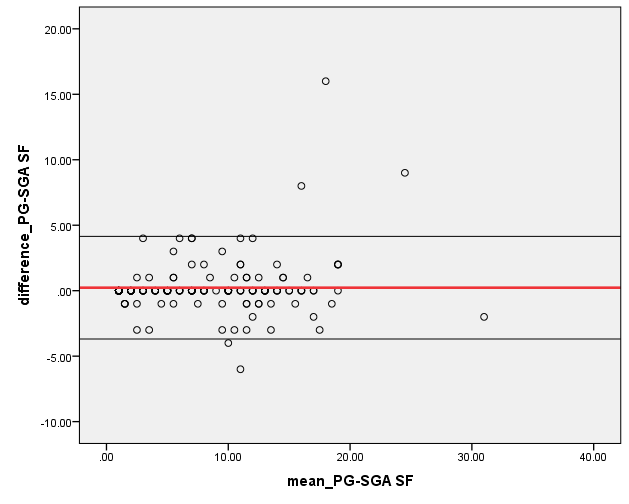 | |  |  |  |  |  |  |  |  |  |  |  |  |  |  |  |  |  |  |  |  |  |  |  |  |  |  | |  | |
|  | |  |  |  |  |  |  |  |  |  |  |  |  |  |  |  |  |  |  |  |  |  |  |  |  |  |  | |  | |
|  | |  |  |  |  |  |  |  |  |  |  |  |  |  |  |  |  |  |  |  |  |  |  |  |  |  |  | |  | |
|  | |  |  |  |  |  |  |  |  |  |  |  |  |  |  |  |  |  |  |  |  |  |  |  |  |  |  | |  | |
|  | |  |  |  |  |  |  |  |  |  |  |  |  |  |  |  |  |  |  |  |  |  |  |  |  |  |  | |  | |
|  | |  |  |  |  |  |  |  |  |  |  |  |  |  |  |  |  |  |  |  |  |  |  |  |  |  |  | |  | |
|  | |  |  |  |  |  |  |  |  |  |  |  |  |  |  |  |  |  |  |  |  |  |  |  |  |  |  | |  | |
|  | |  | |  | |  | |  | |  | |  | |  | | | |  | | |  | |  | |  | |  | |  | |
|  | |  | |  | |  | |  | |  | |  | |  | | | |  | | |  | |  | |  | |  | |  | |
|  | | |  | |  | |  | |  | |  | |  | | | |  | | |  | |  | |  | |  | |  | |  |
|  | |  | |  | | | | | | | | | | | | | | | | | | | | | | |  | |  | |
|  | |  | |  |  |  |  |  |  |  |  |  |  |  |  |  |  |  |  |  |  |  |  |  |  |  |  | |  | |
|  | |  | |  |  |  |  |  |  |  |  |  |  |  |  |  |  |  |  |  |  |  |  |  |  |  |  | |  | |
|  | |  | |  |  |  |  |  |  |  |  |  |  |  |  |  |  |  |  |  |  |  |  |  |  |  |  | |  | |
|  | |  | |  |  |  |  |  |  |  |  |  |  |  |  |  |  |  |  |  |  |  |  |  |  |  |  | |  | |
|  | |  | |  |  |  |  |  |  |  |  |  |  |  |  |  |  |  |  |  |  |  |  |  |  |  |  | |  | |
|  | |  | |  |  |  |  |  |  |  |  |  |  |  |  |  |  |  |  |  |  |  |  |  |  |  |  | |  | |
|  | |  | |  |  |  |  |  |  |  |  |  |  |  |  |  |  |  |  |  |  |  |  |  |  |  |  | |  | |
|  | |  | |  |  |  |  |  |  |  |  |  |  |  |  |  |  |  |  |  |  |  |  |  |  |  |  | |  | |
|  | |  | |  |  |  |  |  |  |  |  |  |  |  |  |  |  |  |  |  |  |  |  |  |  |  |  | |  | |
|  | |  | |  |  |  |  |  |  |  |  |  |  |  |  |  |  |  |  |  |  |  |  |  |  |  |  | |  | |
|  |  | | | | | | | | | | | | | |  |  | | |  |  |  |  |  |  |  |  |  |  |  |  |
|  |  |  |  |  |  |  |  |  |  |  |  |  |  |  |  |  | | |  |  |  |  |  |  |  |  |  |  |  |  |
|  |  |  |  |  |  |  |  |  |  |  |  |  |  |  |  |  | | |  |  |  |  |  |  |  |  |  |  |  |  |
|  |  |  |  |  |  |  |  |  |  |  |  |  |  |  |  |  | | |  |  |  |  |  |  |  |  |  |  |  |  |
|  |  |  |  |  |  |  |  |  |  |  |  |  |  |  |  |  | | |  |  |  |  |  |  |  |  |  |  |  |  |
